# Preventive transarterial embolization in upper nonvariceal gastrointestinal bleeding

**DOI:** 10.1186/s13017-016-0114-1

**Published:** 2017-01-13

**Authors:** Aleksejs Kaminskis, Aina Kratovska, Sanita Ponomarjova, Anna Tolstova, Maksims Mukans, Solvita Stabiņa, Raivis Gailums, Andrejs Bernšteins, Patricija Ivanova, Viesturs Boka, Guntars Pupelis

**Affiliations:** 1 Department of General and Emergency Surgery, Riga East University Hospital, 2 Hipokrata Str. Riga, Riga, LV 1038 Latvia; 2 Department of Interventional Radiology, Riga East University Hospital, Riga, Latvia; 3 Riga East University Hospital, Riga, Latvia; 4 Surgical Department, Riga East University Hospital, Riga, Latvia

**Keywords:** Nonvariceal upper gastrointestinal bleeding, Transarterial, Preventive embolization

## Abstract

**Background:**

Transarterial embolization (TAE) is a therapeutic option for patients with a high risk of recurrent bleeding after endoscopic haemostasis. The aim of our prospective study was a preliminary assessment of the safety, efficacy, and clinical outcomes following preventive TAE in patients with non-variceal acute upper gastrointestinal bleeding (NVUGIB) with a high risk of recurrent bleeding after endoscopic haemostasis.

**Methods:**

Preventive visceral angiography and TAE were performed after endoscopic haemostasis on patients with NVUGIB who were at a high risk of recurrent bleeding (PE+ group). The comparison group consisted of similar patients who only underwent endoscopic haemostasis, without preventive TAE (PE− group). The technical success of preventive TAE, the completeness of haemostasis, the incidence of rebleeding and the need for surgical intervention and the main outcomes were compared between the groups.

**Results:**

The PE+ group consisted of 25 patients, and the PE− group of 50 patients, similar in age (median age 66 vs. 63 years), gender and comorbid conditions. The ulcer size at endoscopy was not significantly different (median of 152 mm vs. 127 mm). The most frequent were Forest II type ulcers, 44% in both groups. The distribution of the Forest grade was even. The median haemoglobin on admission was 8, 2 g/dl vs. 8,7 g/dl, *p* = 0,482, erythrocyte count was 2,7 × 10^12^/L vs. 2,9 × 10^12^/L, *p* = 0,727. The shock index and Rockall scores were similar, as well as and transfusion – on average, four units of packed red blood cells for the majority of patients in both groups, however, significantly more fresh frozen plasma was transfused in the PE− group, *p* = 0,013. The rebleeding rate was similar, while surgical treatment was needed notably more often in the PE- group, 8% vs. 35% accordingly, *p* = 0,012. The median ICU stay was 3 days, hospital stay – 6 days vs. 9 days, *p* = 0.079. The overall mortality reached 20%; in the PE+ group it was 4%, not reaching a statistically significant difference.

**Conclusion:**

Preventive TAE is a feasible, safe and effective minimally invasive type of haemostasis decreasing the risk of repeated bleeding and preparing the patient for the definitive surgical intervention when indicated.

## Background

Rebleeding is one of the most serious complications of endoscopic haemostasis in patients with NVUGIB. The prevention of rebleeding is, therefore, crucial in the treatment of NVUGIB due to a considerable increase in mortality in case of failure.

Over the past two decades, TAE has become the first-line therapy for the management of upper gastrointestinal bleeding that is refractory to endoscopic haemostasis [1]. Despite conservative medical treatment or endoscopic intervention severe rebleeding occurs in 5−10% of patients, requiring surgery or TAE [2], and TAE has been increasingly used as an alternative to surgery in the upper NVUGIB refractory to endoscopic therapy. The method has been associated with a lower mortality and complication rate compared to surgery [3, 4]. Although early aggressive endoscopic haemostasis is generally the first choice of treatment in the cohort of patients who are at a high risk of rebleeding, additional methods of haemostasis may be needed to achieve a favourable outcome. Due to former evidence that in the situation of acute bleeding, it is not always possible to perform TAE successfully as an additional method of haemostasis, the preventive mode of TAE was proposed as one of the possible ways to achieve complete haemostasis, decrease the rebleeding rate, the need for surgical intervention, complications and mortality. A possible benefit of using TAE as a preventive measure in patients who are considered to have a high risk of rebleeding after endoscopic haemostasis has never been properly examined. One of the main arguments in favour of preventive TAE is speculation that rebleeding after a temporarily successful endoscopic therapy might be caused by an inadequate endoscopic treatment resulting in a residual arterial flow beneath the ulcer. In this subgroup of patients, preventive TAE performed shortly after endoscopic haemostasis is achieved, could result in a decreased rate of rebleeding and reduced mortality thereby [5]. The aim of our prospective study was a preliminary assessment of the safety, efficacy, and clinical outcomes following preventive TAE in NVUGIB patients with a high risk of recurrent bleeding after endoscopic haemostasis.

## Methods

**Fig. 1:**
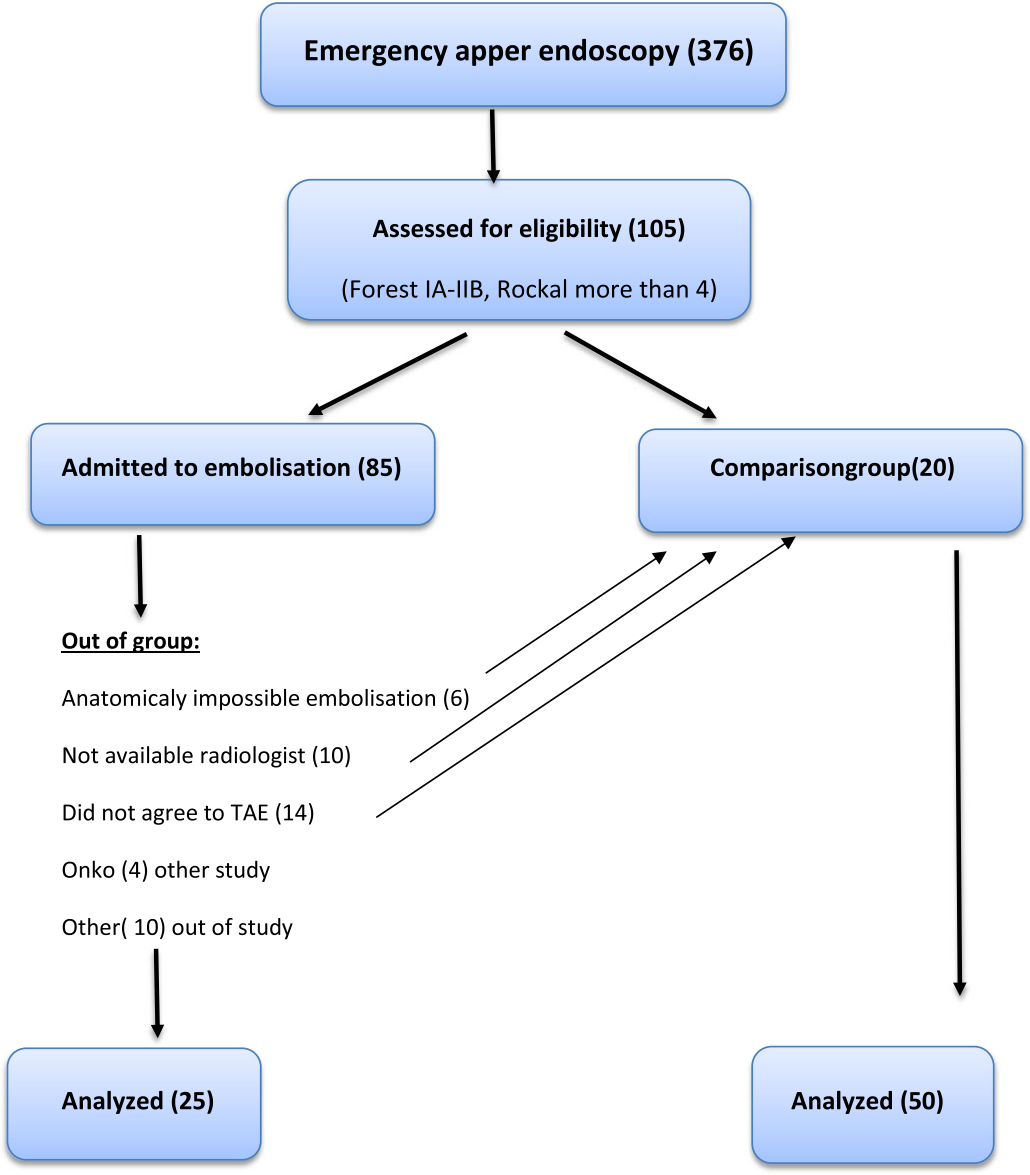
Patient recruitment chart

The preparation of the study included an analysis of the medical charts of 379 patients who were emergently admitted to the Riga East University Hospital with NVUGIB in the period from 2010 to 2013, (Fig. 1). The results suggested that the patients with NVUGIB who were at a high risk of rebleeding after emergent endoscopic haemostasis had Forrest I-IIb type of ulcer and the Rockall score ≥ 5. These two criteria were important for further grouping and inclusion of patients in the prospective study. Informed consent was obtained from the patients who underwent endovascular treatment. Preventive visceral angiography and TAE were performed on patients with acute NVUGIB who were considered to be at a high risk of recurrent bleeding after endoscopic haemostasis according to the evidence of Forest I-IIb ulcer and Rockall score ≥ 5 (PE+ group). The comparison group consisted of similar patients who underwent only endoscopic haemostasis, patients who did not agree to undergo preventive TAE with a similar prognosis of high rebleeding risk after endoscopic hemostasis and similar comorbid conditions (PE− group). The exclusion criterion was terminal end stage renal disease. The participants were enrolled and assigned to their treatment by the consensus among the consultant surgeon, consultant radiologist and duty endoscopy specialist. Endoscopic combination therapy (injection of diluted adrenaline 1:10,000, treatment with a heater probe, and/or hemoclip) followed by a 72-h infusion of esomeprazole (80 mg bolus followed by 8 mg/h) was applied to all patients. Blood transfusion was given if haemoglobin was lower than 9.7 g/dl. Patients were closely monitored at ICU.

### Technical approach

The technical goal was preventive embolization of the left gastric artery or the gastroduodenal artery (depending on the ulcer localization) within 24 h of endoscopic haemostasis, achieving a decrease of the arterial flow in the tissue beneath the ulcer. Usually, proximal embolization of the left gastric artery with coil and/or sandwich type TAE embolization of the gastroduodenal artery was used. In cases with the ulcer localized in the smaller or greater curvature or the gastric fundus – the left gastric artery was obliterated; in gastric antral, pyloric or duodenal ulcers – the gastroduodenal artery was embolized. Rebleeding was defined as a presence of hematemesis, blood from the nasogastric tube, or melena associated with a fall in haemoglobin of more than 0.8 g/dl (not explained by hemodilution) or arterial hypotension after primary endoscopy. If the therapeutic endoscopy were insufficient to comparison haemorrhage (technically difficult primary therapeutic endoscopy or signs of exsanguination), TAE or surgical haemostasis could be performed without being preceded by repeated endoscopy. The complication rate, recurrence of bleeding, and the need for repeat endoscopic therapy or surgery were the variables for the statistical analysis in groups. In-hospital mortality rate among the groups was analysed, and the patients who were excluded from the study. The study was approved by the local research ethics committee and followed the declaration of Helsinki [Helsinki declaration]. All authors had access to the study data and have reviewed and approved the final manuscript.

## Results

During the 32-month period of inclusion 75 patients received endoscopic haemostasis for acute high-risk NVUGIB and were evaluated according to the inclusion criteria. The PE+ group consisted of 25 patients, and the PE− group of 50 patients. The median age of patients was 66 years (IQR 74–57) vs. 63 (IQR 75–52) years without a statistically significant difference. There was no difference in gender and comorbid conditions, including the presence of cancer, liver and pulmonary diseases, which were rare in both groups, Table 1. Endoscopic findings were not different, with the median ulcer size of 152 mm (IQR 400–79) vs. 127 mm (IQR 225–79). The bleeding site was similar in both groups. Gastric ulcer was the cause of bleeding in 52% of patients from the PE + group, and 54% of patients from the PE− group, *p* = 0,870. Duodenal ulcer was the cause of bleeding in 48 and 46% of patients respectively. The most commonly found ulcers were Forest II type in 44% of cases in both groups. The distribution of the Forest grade was even, Table 1. The median haemoglobin level on admission was 8,2 g/dl (IQR 11–7) vs. 8,7 g/dl (IQR 10–5,8), *p* = 0,482 and the erythrocyte count was 2,7 × 1012/L (IQR 3,5–2,1) vs. 2,9 × 1012/L (IQR 3,7–2,1), *p* = 0,727 including similar shock index and Rockall scores for both groups. The Rockall score values and shock index values were evenly distributed in the groups, Table 2. The use of anticlotting drugs in the groups was also similar, Table 3. Transfusion support was needed for the majority of patients, using, on average, four units of packed red blood cells (PRBC) in both groups and significantly more fresh frozen plasma (FFP) in the PE− group, *p* = 0,013. The rebleeding rate was similar, however, surgical treatment in patients who did not undergo embolization was needed significantly more often, 8% vs. 35,4%, *p* = 0,012. The median ICU stay was 3 days, and the hospital stay did not differ either, 6 (IQR 10–6) vs. 9 (IQR 11–6), *p* = 0,079. Mortality for all patients reached 20%, however, in the PE+ group it was 4%, though not reaching a statistically significant difference. The main outcomes are displayed in Table 4.

**Table 1:** Patient characteristics

Variables	PE+ *n* = 25	PE− *n* = 50	*p*
Age, years, median (IQR)	66 (74-57)	63 (75-52)	0,393
Gender/Male, no. of patients	16 (64%)	34 (68%)	0,797
Comorbidities, no. of patients	18 (72%)	35 (70%)	0,858
Heart disease, no. of patients	13 (52%)	30 (60%)	0,509
Kidney disease, no. of patients	3 (12%)	6 (12%)	1,000
Liver disease, no. of patients	2 (8%)	4 (8%)	1,000
Cancer, no. of patients	1 (4%)	1 (2%)	1,000
Metabolic disease, no. of patients	4 (16%)	10 (20%)	0,763
Respiratory disease, no. of patients	2 (8%)	4 (8%)	1,000
Cerebral disease, no. of patients	6 (24%)	11 (22%)	0,881
Ulcer size, mm, median (IQR)	152 (400-79)	127 (225-79)	0,737
Forrest IA, no. of patients	5 (20%)	11 (22%)	0,937
Forrest IB, no. of patients	4 (16%)	7 (14%)	0,937
Forrest IIA, no. of patients	11 (44%)	22 (44%)	0,937
Forrest IIB, no. of patients	5 (20%)	10 (20%)	0,937
HGB, g/dl, median (IQR)	8,2 (11-7)	8,7 (10-5,8)	0,482
ERY, ×10^12^/L, median (IQR)	2,7 (3,5-2,1)	2,9 (3,7-2,1)	0,727
INR, ratio, median (IQR)	1,07 (1,25-1)	1,16 (1,34-1)	0,318
Shock index, median (IQR)	0,93 (1,2-0,67)	0,86	0,567
Rockall score, points, median (IQR)	6 (5-7)	6	0,608

**Table 2:** Median Rockall score and shock index

Scores	PE+ *n* = 25	PE− *n* = 50	*p*
Rockall score 3	3/12%	7/14%	1.000
Rockall score 4–5	4/16%	11/22%	0.761
Rockall score 6–7	13/52%	21/42%	0.412
Rockall score 8–9	4/16%	10/20%	0.763
Rockall score 10–11	1/4%	1/2%	1.000
Shock index 0,1 >	0/0	3/6%	0.546
Shock index 0,5 >	6/24%	12/24%	1.000
Shock index 0,7 >	9/36%	15/30%	0.600
Shock index 1 >	10/40%	20/40%	1.000

**Table 3:** Anticlotting agents

Drugs	PE+ *n* = 25	PE− *n* = 50	*p*
Anticoagulants	2/8%	5/10%	1.000
Antiaggregants	6/24%	13/26.5%	0.814
NSAID (excluding Aspirine)	3/12%	12/24%	0.359

**Table 4:** Main outcomes

Outcomes	PE+	PE−	*p*
ICU needed	25 (100%)	40 (82%)	0,024
ICU stay, days, median (IQR)	3 (3-2)	3 (5-2)	0,352
Transfusion needed, no of patients	22 (88%)	40 (80%)	0,524
PRBC, units	4 (5-3)	4 (5-2)	0,399
FFP, units, (IQR)	2 (2-2)	3 (4-2)	0,013
Surgery, no of patients	2 (8%)	17 (35%)	0,012
Re-bleeding, no of patients	3 (12%)	11 22,4%	0,358
Hospital stay, days (IQR)	6 (10-6)	9 (11-6)	0,079
Mortality, no of patients	1 (4%)	8 16,3%	0,258

## Discussion

Management of the upper gastrointestinal bleeding is still a challenge despite the recognized leading role of endoscopic hemostasis [6]. All improvements in the medical and endoscopic treatment are not sufficiently effective in treating the aging population with comorbid conditions that often has concomitant treatment with nonsteroidal anti-inflammatory or anti-clotting drugs [1]. The risk of rebleeding increases when Forest I-II ulcers diagnosed during endoscopic hemostasis is associated with different comorbid conditions and medication that interferes with the clotting system [7–9]. When rebleeding happens, several options are recommended – emergent repeated endoscopy, emergent TAE or surgical intervention [7, 10]. Preventive TAE is an additional option to decrease the rebleeding rate after an endoscopic hemostasis attempt. Nevertheless, the exact criteria for the selection of indications and strong evidence supporting the rational to use this method of hemostasis are insufficient [11]. Advances in catheter-based techniques and newer embolic agents, as well as the recognition of the efficiency of minimally invasive treatment options have expanded the role of interventional radiology in the management of hemorrhage for a variety of indications, such as peptic ulcer bleeding, malignant diseases, hemorrhagic Dieulafoy lesions and iatrogenic or trauma bleeding [1, 6]. The technical aspects of TAE in the current study were performed following the recommendations described in the publication of Scandinavian authors, providing diagnostic angiography within 24 h of endoscopic hemostasis. The branches of the celiac trunk and the superior mesenteric artery, and the artery related to the peptic ulcer were identified according to the hemoclip placed during the endoscopic procedure. Microcatheter technique and coils were used routinely, and the blind technique in exclusive cases only [12].

### Risk assessment

The assessment of the medical charts of the patients with acute NVUGI bleeding who were admitted to our institution revealed a group of patients who experienced rebleeding episodes after emergent endoscopic haemostasis. The most frequent endoscopic finding in this cohort was Forrest I-IIb type ulcers and the Rockall score ≥ 5. A further prospective study confirmed this observation complying with the reports from literature including the observation that the patient’s age of over 60 years may be a risk factor. The other criteria, except for blood pressure and heart rate, like haemoglobin levels are reported as insufficiently sensitive in determining the severity of the upper gastrointestinal bleeding [13, 14]; however, patients from both groups who experienced rebleeding in our study had a median haemoglobin level of 8.2–8.7 g/dl before repeated haemostasis. The majority of reports emphasize that patients undergoing a transarterial procedure for the evaluation and management of haemorrhage are often poor surgical candidates due to the hemodynamic instability, comorbid conditions and coagulopathy [1, 7, 8]. In the current study, the patient condition in both groups was quite similar before repeated haemostasis, including the incidence of comorbid conditions, level of blood loss, shock index and coagulation status complying with the recommendations [1, 7, 8]. The total need for transfusion was not different among the groups, including the transfused amount of PRBC units, while significantly more FFP units were needed for patients who did not undergo preventive TAE. The strategy was preventive and the decision to perform prophylactic preventive TAE was the decision of the duty personnel in consensus with the consultant surgeon, consultant radiologist and duty endoscopy specialist. This strategy allowed to perform preventive TAE shortly after the inclusion criteria were established and indirectly supported the evidence that early TAE is associated with lower morbidity and mortality [2, 15, 16]. It is difficult to pinpoint the author of the idea of preventive embolization, however, a large part of clinical data and tactical recommendations are published by Scandinavian authors [1, 2, 15, 16]. The current study supports the reported positive experience with preventive TAE. Both patient groups were at a high risk of rebleeding after endoscopic hemostasis, and even if the ICU and hospital stay was not different, the need for surgical intervention was significantly higher in patients who did not undergo preventive TAE. Even more, mortality was also higher, albeit not significantly, probably due to a small sample size. The weak points of our study were the relatively small sample size and unpowered statistics. All the same, as in other reports, the uncertain selection of criteria for inclusion and the need for a larger cohort of patients were the weaknesses of the study. The advantages included the availability of the duty stuff for the definition of the inclusion criteria and the rather short time span for performing preventive TAE.

## Conclusion

Preventive TAE is a feasible, safe and effective minimally invasive type of haemostasis, decreasing the risk of repeated bleeding and preparing the patient for the definitive surgical intervention when indicated. The availability of the duty stuff and the performance of TAE soon after the indications are defined increase the efficiency of the procedure.

## Abbreviations

ICU: Intensive care unit
IQR: Inter quartile range
NVUGIB: Nonvariceal upper gastrointestinal bleeding
PE: Preventive embolization
TAE: Transcatheter arterial embolization
